# Supplementary material for: Molecular Cytogenetics Reveals Mosaicism in Human Umbilical Vein Endothelial Cells
Source: Genes (Basel). 2022 Jun 3;13(6):1012. doi: 10.3390/genes13061012 (PMC9222953; doi:10.3390/genes13061012)
Supplement: Supplementary file 1 [file genes-13-01012-s001.zip › genes-1741271-supplementary.pdf]

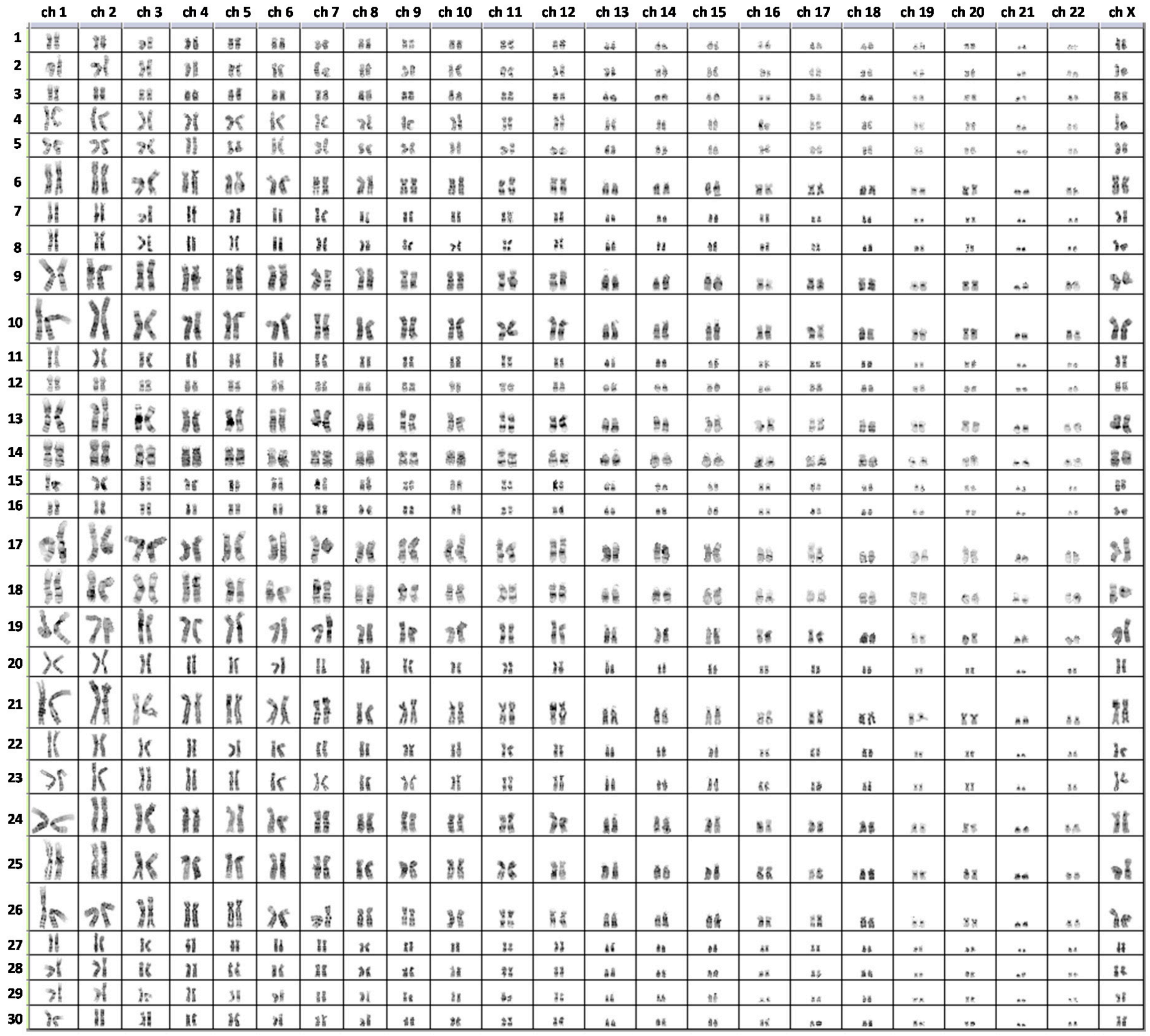

Supplemental Figure S1. Comparison of 30 karyograms of HUVECs analyzed by G-banding.

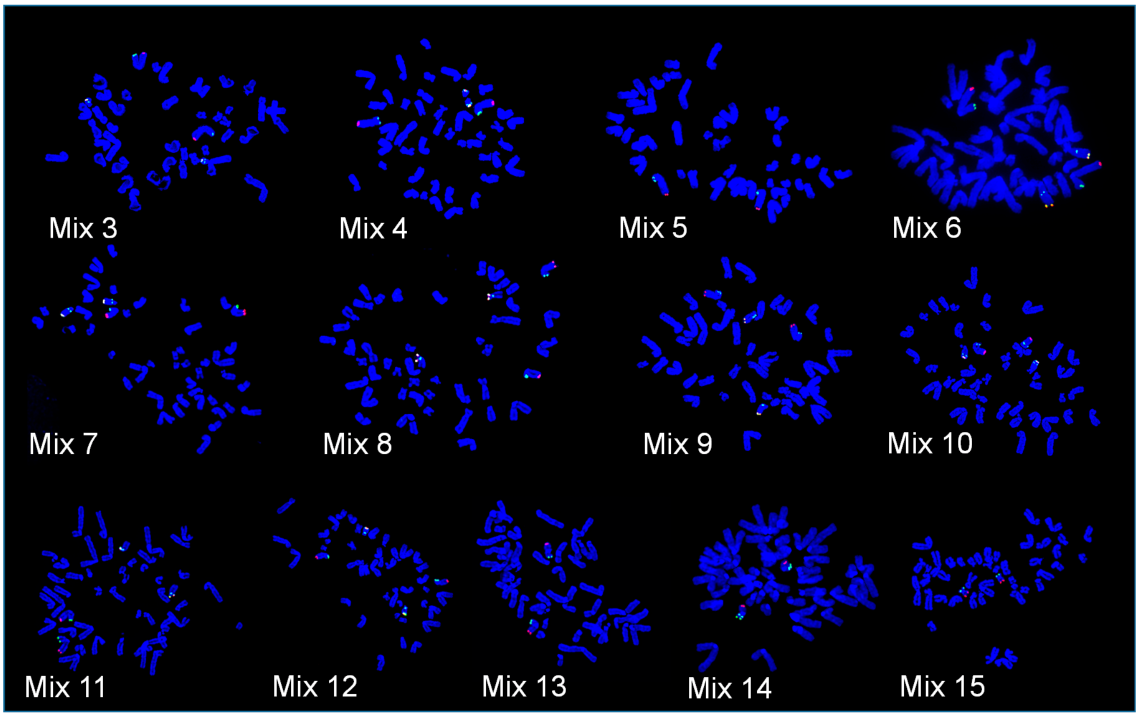

Supplemental Figure S2. Signal patterns for ToTelVysion mixes 3-15 (See Table 1 for the details)

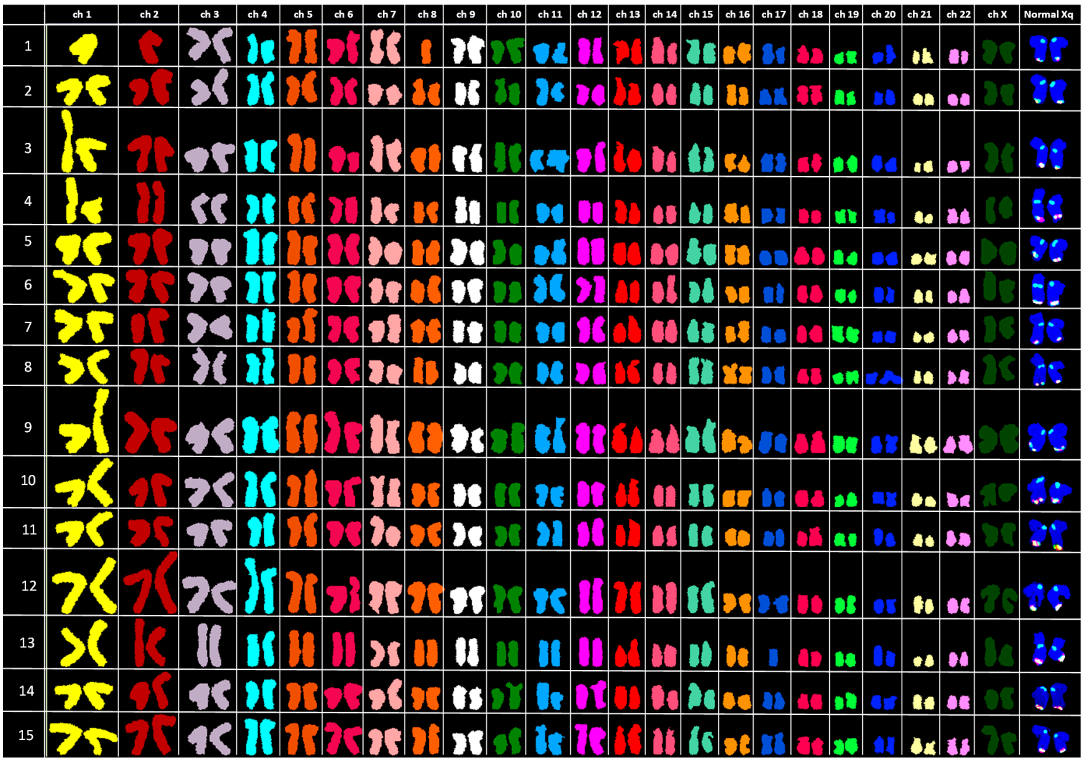

Supplemental Figure S3. Comparison of 15 classified SKY cells with subsequent hybridization using ToTelVysion mix 2 showing a normal Xq ter.

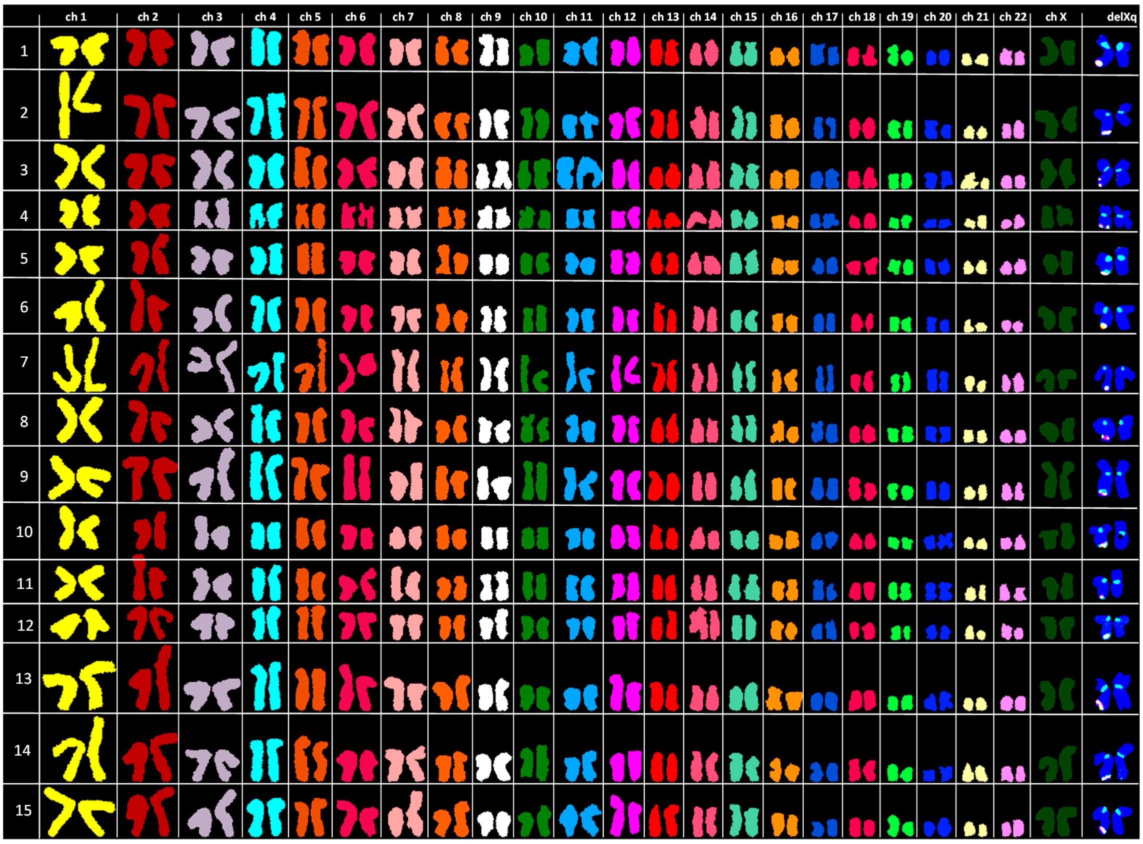

Supplemental Figure S4. Comparison of 15 classified SKY cells with subsequent hybridization with ToTelVysion mix 2 showing a deletion of one Xq homologue in each cell.
